# A History of Dystonia: Ancient to Modern

**DOI:** 10.1002/mdc3.12493

**Published:** 2017-05-22

**Authors:** Rachel E. Newby, Deborah E. Thorpe, Peter A. Kempster, Jane E. Alty

**Affiliations:** ^1^ Neurosciences Department Monash Medical Centre Melbourne Victoria Australia; ^2^ Department of Neurology Leeds General Infirmary Leeds United Kingdom; ^3^ Hull York Medical School University of York York United Kingdom; ^4^ Centre for Medieval Studies University of York York United Kingdom; ^5^ Centre for Chronic Diseases and Disorders (C2D2)/Electronics Department University of York York United Kingdom; ^6^ Department of Medicine Monash University Melbourne Victoria Australia

**Keywords:** art, dystonia, history, literature, psychiatry

## Abstract

Before 1911, when Hermann Oppenheim introduced the term dystonia, this movement disorder lacked a unifying descriptor. While words like epilepsy, apoplexy, and palsy have had their meanings since antiquity, references to dystonia are much harder to identify in historical documents. Torticollis is an exception, although there is difficulty distinguishing dystonic torticollis from congenital muscular torticollis. There are, nevertheless, possible representations of dystonia in literature and visual art from the pre‐modern world. Eighteenth century systematic nosologists such as Linnaeus, de Sauvages, and Cullen had attempted to classify some spasmodic conditions, including torticollis. But only after Charcot's contributions to clinical neuroscience were the various forms of generalized and focal dystonia clearly delineated. They were categorized as *névroses*: Charcot's term for conditions without an identifiable neuroanatomical cause. For a time thereafter, psychoanalytic models of dystonia based on Freud's ideas about unconscious conflicts transduced into physical symptoms were ascendant, although there was always a dissenting “organic” school. With the rise of subspecialization in movement disorders during the 1970s, the pendulum swung strongly back toward organic causation. David Marsden's clinical and electrophysiological research on the adult‐onset focal dystonias was particularly important in establishing a physical basis for these disorders. We are still in a period of “living history” of dystonia, with much yet to be understood about pathophysiology. Rigidly dualistic models have crumbled in the face of evidence of electrophysiological and psychopathological overlap between organic and functional dystonia. More flexible biopsychosocial frameworks may address the demand for new diagnostic and therapeutic rationales.

Without the marker of nomenclature, it can be hard for the modern reader to catch sight of the historical traces of a disease. Even movement disorders that must have had a visible presence in past societies lack focus in written accounts until a framework of knowledge is created around a name. The shaking palsy—common and easily observable—was poorly recognized before James Parkinson (1755–1824) made sense of the fragmentary classifications of tremor in 1817. The term dystonia was not introduced until 1911, when Hermann Oppenheim (1858–1919) described the sustained spasm of dystonia musculorum deformans.^1^ Athetosis (without fixed posture) was first used just 40 years earlier for more dynamic movements that would satisfy a modern definition of hemidystonia.^2^ Torticollis has a longer tradition in medical writing, although there is difficulty distinguishing dystonic torticollis from congenital muscular torticollis caused by sternocleidomastoid injury. There are possible representations of dystonia in visual art and literature that predate coherent medical descriptions by several centuries.

When, during the late 19th and early 20th century, a body of knowledge about dystonia was being assembled, two intellectual undercurrents were shaping the development of modern neurology. Both had a lasting effect on thinking about dystonia. With his *methode clinico‐anatomique*, Jean‐Martin Charcot (1825–1893) created a separation of “organic” disorders, which could be matched to structural changes in the nervous system, from “functional” disorders, which could not. As various forms of dystonia were delineated over the next couple of decades, they were classified as *névroses*: his term for conditions without an identifiable neuroanatomical cause. Although Charcot, at least for a time, thought that even hysteria might come under the organic umbrella, his career hastened the dichotomy of neurology and psychiatry. The second influence was Sigmund Freud's (1856–1939) theories about early life experiences and resultant psychic distress transformed into physical symptoms. These were soon applied to dystonia, which for many years occupied a shadowy territory between neurology and psychiatry.

This is a review of dystonia's history from possible depictions in ancient art and literature up to recent classifications based on neurogenetics. The past still has its influences on this movement disorder, with uncertainties about how psychological factors are operating in both organic and functional (psychogenic) dystonia, and the legacy of mind‐brain dualistic thinking about pathophysiology.

## Sources from the Ancient World

References to spasmodic cervical conditions in the writings of physicians of the ancient world are rather obscure. Hippocrates’ *traxhlos sklhros* means “a stiff and painful neck,” a fatal sign when accompanied by “contraction of the jaws, a powerful throbbing of the jugular vessels, and contraction of the tendons.”^3^, ^4^ He may have been referring to tetanus or meningitic neck stiffness rather than torticollis, and Celsus later used the term *rigor cervicis* in a similar context.^5^ Celsus also described a rigor of the sinews, *rigore nervorum*, that “draws down the head to the shoulder‐blades, now the chin to the chest, now stretches out the neck straight and immobile.”^6^ Pliny the Elder, who was not a physician, made mention of *rigor cervicis*, along with a number of suggested remedies for softening (*mollitur*) the neck.^7^

The abnormal neck posture seen in some statues of Alexander the Great has prompted the suggestion that he had ocular torticollis.^8^ This is probably an over‐interpretation, since not all statues depict this posture, and texts describing his appearance are ambiguous in their original languages.^9^ Plutarch claimed that the sculptor Lysippus had “accurately observed” Alexander's neck, which “was bent slightly to the left.”^10^ Plutarch wrote this centuries after Alexander's death, and it is not clear on what source he based his assertion.^11^

The 2000‐year old ceramic sculptures of the Moche civilization of Peru render many diseases and deformities. Martinez‐Castrillo et al. have argued that the horizontally tensed lips and pronounced nasolabial folds of one such sculpture represents the first depiction of Meige syndrome.^12^ This speculation is given some support from the attention of the Moche to individual differences in their sculpture, with various facial disfigurements that appear to portray cutaneous leishmaniasis, ritual mutilation, and cleft lip.^13^

## Medieval and Renaissance Depictions

**Figure 1:**
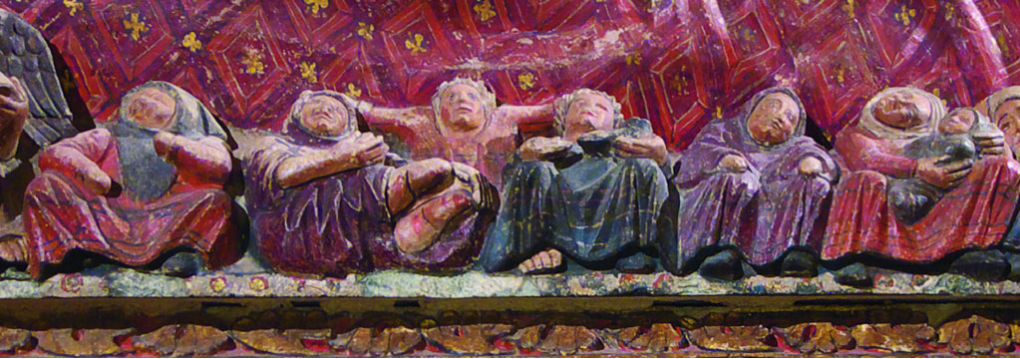
Abnormal neck positions depicted on the tomb of the medieval Bishop Pedro de Osma (1040–1109), Cathedral of Burgo de Osma, Spain. These carvings were executed c.1258. Photograph by José Luis Filpo Cabana.

Disabilities often appear in medieval religious art, both in the illustrated margins of manuscripts and in churches. A set of painted figures from El Burgo de Osma cathedral in Spain look as if they have cervical dystonic postures, with heads tilted to the left and pained facial expressions (Fig. 1).^9^ There is the usual difficulty in differentiating dystonic from muscular torticollis. Indeed, the muscular form might have been more frequent in the Middle Ages because of obstetric injury. Certain medieval texts record postures that are more consistent with muscular torticollis; for example, one of the 12th‐century miracles of William of Norwich concerns an 8‐year‐old girl described as having a twisted neck (*ceruicem contractis*), so that her left cheek was touching her shoulder. The miracle describes how the neck could not be bent in any direction without also bending the shoulder.^14^, ^15^ Nicole Oresme (1320–1382), in his *Quaestio contra divinatores horoscopios*, noted that the necks of those suffering from melancholy were suddenly thrust or twisted backwards (*habebit caput vel collum retortum*), causing marvel‐like experiences.^16^

In the Middle Ages, scribes reported occupational disturbances of writing; a widespread note was *Dextera scriptoris careat gravitate doloris* (The scribe's right hand shall be liberated from the gravity of pain).^17^ Convincing material evidence of dystonia affecting handwriting has been found in legal documents written by the French scribe Bernard Blancard between 1297 and 1343.^18^ The dated documents preserve the chronology of a progressive deterioration in Blancard's script, with a multi‐directional, jerky tremor and probable involvement of hand posture.

*Pantegruel* by François Rabelais (1494–1553) describes how the beheaded Epistemon was healed. A poultice was applied, “so as not to make him a ‘wry neck’” (*afin qu'il ne fust torty colly*).^19^, ^20^, ^21^ This is the first known use of the word torticollis—in this case, a satirical reference to the head bowing of religious hypocrites—and the term would subsequently be adopted as a medical description.^22^

David Marsden (1938–1998), writing about blepharospasm‐oromandibular dystonia in 1976, suggested it be called Brueghel's syndrome.^23^ He cited *De Gaper* by Pieter Brueghel the Elder (c.1525–1569), which appears to depict involuntary jaw opening and eye closure. Marsden assumed Brueghel had observed this syndrome. Other scholars have been less convinced, seeing the subject caught in mid‐yawn to display the artist's proficiency with facial expression.^24^, ^25^

## 17th and 18th Centuries: The Medical Nosologists

One of the earliest medical records of cervical dystonia, by Swiss physician Felix Platerus (1536–1614), describes “a kind of spasm in which the head was turned to the left side.”^26^

Samuel Kinnier Wilson (1878–1937) used this verse by the poet and dramatist Paul Scarron (1610–1660) as an epigraph to his textbook chapter on torticollis:

*Mon pauvre corps est raccourci* (My poor body is shortened)
*Et j'ai la tête sur l'oreille (*And I have my head on my ear)
*Mais cela me sied à merveille* (But it suits me marvelously)
*Et parmi les torticollis* (And among the stiff‐necked)
*Je passe pour des plus jolis* (I pass for one of the prettiest)^27^, ^28^

Scarron's postures resemble dystonic torticollis. While some paintings and engravings show the poet with his head inclined forward and to the right, there are portraits with a leftward tilt, suggesting variable muscle overactivity.

During the Enlightenment era of the 17th and 18th centuries, physicians began to move beyond the authority of the medical writers of the pre‐modern world. Enlightenment approaches to the natural sciences relied on observation and classification as keys to new knowledge. Thomas Sydenham (1624–1689) had realized that illnesses could be separated into “types” according to their symptoms, visible manifestations, and chronology, similar to the way in which natural things like plants, animals, and rocks could be organized into groupings. The botanical and zoological classification *Systema Naturae*, published in 1735 by Carl Linnaeus (1707–1778), revolutionized the study of living things by ordering them into hierarchies of Class, Order, Genus, and Species, with a binomial genus‐species nomenclature. Linnaeus was a medical practitioner, and he also produced a “Linnaean” disease nosology, his *Genera Morborum*, although it was less successful than *Systema Naturae* as a work of classification. He gave a class of MOTORII for involuntary movement, under which he listed spasmodic conditions, and also a genus Hieranosos for dynamic movements not otherwise specified. François Boissier de Sauvages (1706–1767), who was a botanist before becoming a physician and was a friend of Linnaeus, referred to spasmodic torticollis under a class SPASMI in his *Methodical Nosology* (1763). William Cullen's (1710–1790) *System of Nosology* introduced a class of NEUROSES that contained many nervous system disorders. In a genus Contractura, he lists the species obstipas spasmodica, which equated to torticollis.

The systematic 18th century nosologies—those of Linnaeus, Vogel, Sauvages, and Cullen—were vertical lineages created by differential characterization. The presupposition that medical species, the building blocks of these taxonomic rankings, were as distinct as plant and animal species was their weakness, and these listings now look archaic. They leave their mark on neurology in the continued use of Latin binomials for a few diseases and in a tradition for collecting and categorizing clinical information.

## The 19th and Early 20th Century: Dystonia's Classifications

### Early Onset Generalized Dystonia

Oppenheim introduced the term dystonia muscularum deformans to describe abnormal posturing in 4 unrelated Jewish children.^1^ The Polish neurologists Flatau and Sterling published similar observations in the same year.^29^ Oppenheim's belief that the syndrome had an organic basis stood in opposition to that of Schwalbe (1883–1927), whose earlier account of “torsion neurosis” in 3 siblings strongly emphasized hysterical features.^30^ Two case reports written by Destarac in 1902, largely overlooked at the time, gave a detailed account of the condition without settling on a unifying descriptive term. Like Oppenheim, he believed that it had an organic basis.^31^

### Idiopathic Focal Dystonia

The idiopathic focal dystonias—blepharospasm; oromandibular, cervical, and laryngeal dystonias; and the various occupational dystonias—were once treated as independent nosological entities. Phenomenological features such as task‐specificity and relief with voluntary maneuvers were considered inconsistent with organic disease. An intimate relationship with social stress, the mostly female predominance, and a correlation with certain personality traits further supported psychogenicity.

#### Cervical Dystonia

In 1888, Charcot presented a case of *spasme clonique du sterno‐mastoïdien et du trapeze* in a stockbroker. The spasms started after catastrophic personal financial loss, establishing cervical dystonia's strong tradition of psychological attribution.^32^ Edouard Brissaud (1852–1909) labeled it “torticollis mental”—opining a psychogenic cause on the basis that abnormal posturing might be extinguished by a light touch to the head. He dismissed this sign as “a simple mannerism, or childish behavior or pathological fake.”^33^ Brissaud's pupils Henry Meige (1866–1940) and Louis Feindel (1862–1930) coined the term *geste antagoniste efficace* and wrote about its psychological causes.^34^ William Gowers (1845–1915) did allow that there might be a “true” form resulting from overactivation of lower brain centers, as well as hysterical torticollis. Joseph Babinski (1857–1932) reported two cases of coincident neck and upper limb spasm and hypothesized corticospinal pathology.^35^ A 1907 monograph by René Cruchet (1875–1959) divided 357 cases of spasmodic torticollis into 7 etiological classes, emphasizing the lack of nosographical specificity.^36^

#### Blepharospasm‐Oromandibular Dystonia

Meige, who called this syndrome *spasme facial median*, discerned a melancholic, introspective temper in many of his patients. His first accounts focused on sufferers’ “lack of psychical equilibrium” and “fecund imagination,” with relapses and remissions following the ebb and flow of emotional stress.^34^ But later, in a 1910 monograph, Meige suggested the cause might be an irritative focus in the pons or midbrain.^37^ Meige's *volte face* on psychological origin stemmed from his observation of torticollis, facial spasm, and writer's cramp in survivors of Von Economo's encephalitis.

#### Laryngeal Dystonia

The first description of dystonia affecting the vocal cords, causing “nervous hoarseness,” is frequently ascribed to Ludwig Traube (1818–1876).^38^ While later authors cited him as evidence that laryngeal dystonia was a psychoneurotic condition, Traube had been more neutral about causation. Several of its quirks perpetuated the categorization as a psychogenic malady. Some patients are able to sing, and others can talk flawlessly in their sleep, although their waking speech is grossly disordered. Onset may be abrupt, with symptoms that worsen with stress.

#### Writer's Cramp (Focal Upper Limb Dystonia)

The earliest medical report of occupational disturbance of writing, from Italian physician Beradino Ramazzini (1633–1714) in 1713, was about muscular fatigue rather than spasm.^39^ The disorder as we now recognize it was originally described by Charles Bell (1774–1842), who encountered an epidemic of writer's cramp in clerks of the British Civil Service in 1830.^40^ Guillaume‐Benjamin Duchenne (1806–1875) was the first to distinguish occupational spasm (*spasme fonctionnel*) from occupational muscle paralysis (*paralysie musculaire fonctionnelle*).^41^ Both Wilhelm Erb (1840–1921) and Moritz Romberg (1795–1873) supported an organic cause.^42^, ^43^ These early theories lost favor, and subsequent outbreaks in telegraphists and typists were regarded as hysterical manifestations in emotionally vulnerable individuals.

#### Other Occupational Dystonias

In reports from the second half of the 19th century, these disorders were referred to variously as “craft palsies,” “occupational neuroses,” or “professional impotence.” The first descriptions of musician's dystonia, from Romberg in 1853 and Bianchi in 1878, were of task‐specific flexion of the digits in a pianist and a flautist.^44^, ^45^ Robert Schumann (1810–1856) may have had this disability. His correspondence describes pain and stiffness in the fingers while playing the piano, spreading to adjacent muscles, and fluctuating with stress levels.^46^, ^47^

## Dystonia in the 20th Century: The Neurology‐Psychiatry Borderland

When neurology diverged from psychiatry after the turn of the 20th century, it retained the dystonias amongst organically unaligned or functional conditions. Progress in pathological and biochemical research slowly reduced the size of this group. In Kinnier Wilson's posthumously published reference text *Neurology* (1940), the list of the motor neuroses had been distilled to a relatively small number, including focal dystonias, tics, and myoclonus.^48^

Psychoanalytical theories of human behavior, which penetrated art, literature, and popular culture in the decades after Freud's writings, came to exert a strong influence on attitudes to dystonia. Theories about underlying oedipal conflicts and psychosexual anxiety appeared, given weight by sporadic reports of relief from dystonia after psychotherapy and the perception that many of these patients had emotionally unstable personalities. Symbolic interpretations of phenomenology were popular: the twisting of the neck in cervical dystonia was thought to represent a turning away from stressful situations, and the forced eye closure of blepharospasm to signify a desire to close one's eyes on the world.^49^ Within these theories, the term *neurosis* took on a different meaning—denoting conditions in which unconscious conflicts or defense mechanisms were transduced into physical symptoms. The rising popularity of these psychoanalytic models and the absence of concordant neuropathology led, in 1929, to the Réunion Neurologique Internationale Annuale consensus that dystonia was not a disease of the nervous system.^26^ Meige's revisionary argument that focal cranial dystonia should be considered a disorder of the basal ganglia^50^ received little support.

Other challenges to the prevailing psychogenic model eventually appeared. The detailed descriptions of torsion dystonia by Ernst Herz (1900–1965) saw generalized dystonia accepted once more as an organic disease in 1944.^51^, ^52^, ^53^ Zeman et al. demonstrated the hereditary nature of dystonia in 1959.^54^ Favorable outcomes were obtained in some patients treated with thalamotomy or pallidotomy,^55^ whereas Eldridge et al. reported on the limited efficacy of psychotherapy.^56^ An animal model of dystonia after basal ganglia lesioning was described by Denny‐Brown in 1965.^57^

The rise of subspecialization in movement disorders during the 1970s brought new approaches to dystonia, and two dominant intellectual figures of the period led the way to its reconceptualization. Stanley Fahn pointed out how past attempts to manage generalized dystonia along psychiatric lines had been wrong‐headed and had ignored good evidence for organic causation.^58^ David Marsden, with a subtle, inventive mind in which clinical understanding was informed by neuroscience, took the adult‐onset focal dystonias out of their indeterminate classification as neuroses.^59^ His argument that these disorders had a physical rather than psychiatric basis had two main threads. He observed that identical patterns of involuntary movement occurred in the setting of unequivocally organic diseases of the basal ganglia—hereditary cases of generalized dystonia; and survivors of encephalitis lethargica. Using electrophysiological techniques, he found common patterns of disturbed agonist‐antagonist muscle activation in dystonia that implied extrapyramidal dysfunction.^60^

For a time, the pendulum swung so far away from psychological modeling of dystonia that any psychiatric symptoms were presumed secondary to the distress created by the involuntary movements. However, it soon became clear that an entity of psychogenic or functional dystonia did exist. The breadth and authority of Marsden's research also eclipsed some impressions about the adult‐onset focal dystonias that older neurologist authors like Gowers and Kinnier Wilson had recorded in their textbooks. Stereotypic presentations—the anxiously ruminating woman with spasmodic torticollis; or the fastidious clerical worker, worn down by years of occupational penmanship, who develops writer's cramp—suggested a blurring in these disorders of the physical‐psychological demarcation.

## Dystonia in 19th and 20th Century Art and Literature

It is interesting to look at some artistic representations that were created around the time that the medical literature about dystonia was taking shape. Advances in medical science during the 19th century had coincided with the development of the novel as the dominant literary form. Influences extended in both directions—the use by writers of medical realism derived from new scientific knowledge, and the recognition by doctors that the narrative methods used in novels could help to organize information in clinical accounts. If correctly interpreted, Charles Dickens (1812–1870) may have been using the novelist's sharp eye for character detail to pick out examples of dystonia on the bustling streets of London, with similarities to James Parkinson's “field neurology” method for the shaking palsy 35 years earlier.

Dickens’ *David Copperfield* (1850) has several possible examples of dystonia—the repeated use of the epithet “writhing” for the malevolent Uriah Heep; and the vain Mr. Sharp, who is described as “carrying his head on one side, as if it were a little too heavy for him.” Mr. Creakle, young David's harsh and dictatorial headmaster, “had no voice, but spoke in a whisper,” suggesting spasmodic dysphonia.^61^ In line with Victorian physiognomy, these grotesque physical attributes were used to call attention to unattractive features of their inner selves.

Raymond Chandler (1888–1959) was one 20th century novelist who used well‐researched medical material to enhance the stories of his detective protagonist Philip Marlowe's seedy, corrupt world. In *The High Window* (1942, later filmed as *The Brasher Doubloon*), he gives a character called Merle Davis spasmodic torticollis (“her head was drawn around to the left about 45 degrees”) conjoined with oromandibular muscle contractions to mark her psychological instability as she reacts to a web of intrigue and criminality.^62^

**Figure 2:**
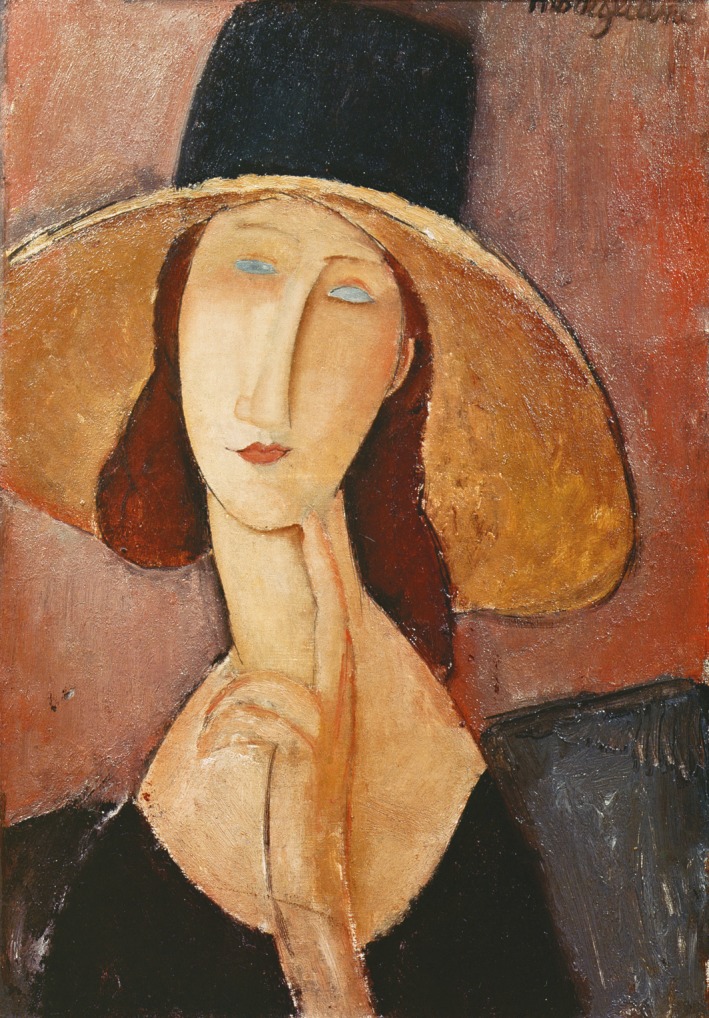
Cervical dystonia in Modigliani's *Jeanne Hébuterne with Large Hat*? Curved facial features accentuate the neck posture as her blank eyes fix the viewer. Kinnier Wilson's translation of Meige and Feindel's book and his subsequent writings crystallized *geste antagoniste* in neurological usage.^64^, ^65^

Dystonia made an appearance in the visual art of the early 20th century. Published photographic images of dystonia and other movement disorders that emanated from the Paris neurological school around this time may have introduced these unusual postures, with their associations of psychological complexity, into artistic practices. Egon Schiele (1890–1918), it has been suggested, used dystonia‐like attitudes as a stylistic element of expressionism, having also been influenced by Freud's ideas about outward projection of psychic conflicts.^63^ Amedeo Modigliani (1884–1920) often evoked sensuousness in portraiture with elongation, curvature, and torsion of upper body parts that resembles dystonia. This is particularly noticeable in paintings of Jeanne Hébuterne, his common‐law wife. The pose shown in Figure 2, with two fingers lightly touching the tilted face, is typical of a sensory trick used in torticollis. There is no hard evidence that she had cervical dystonia, although several photographs of her show head angulation and a hypertrophied right sternocleidomastoid muscle. The day after Modigliani died from tuberculous meningitis, Jeanne jumped from a fifth‐floor window, killing herself and their unborn child.

## Modern Dystonia's Unresolved History

Dystonia's classification system is now informed by neurogenetics. The DYT1 gene, responsible for the majority of early onset generalized dystonia, was localized to chromosome 9 in 1989 and sequenced 8 years later.^66^, ^67^ Since then, over 20 genetically defined dystonia subtypes have been described, and many more inherited degenerative conditions are recognized to possess dystonia as part of their broader phenotype. After international meetings of dystonia experts (Florence in 2009 and Barcelona in 2011), recommendations for the classification of dystonia were generated with categorization arranged along two axes: etiology and clinical presentation.^68^, ^69^ It seems unlikely that this will be the final word on nosology, for the repackaging has not obliterated old fault lines that exist where psychiatric disorders border dystonia. Functional dystonia proved especially hard to classify. It was finally listed as an acquired dystonia, but some questioned whether pseudodystonia might be a more appropriate descriptor.

New knowledge about genetic dystonia shows how blurred the psychiatry‐dystonia boundary really is. There is an excess of psychopathology in these conditions—depression in DYT1, and anxiety spectrum disorders in DYT11 (myoclonus dystonia). The pattern of expression of these psychiatric maladies—present in nonmanifesting carriers and lacking correlation with motor severity—suggests that they are part of dystonia's endophenotype.^70^, ^71^, ^72^, ^73^ Recent case‐controlled research shows an increased prevalence of depression, anxiety, and obsessive‐compulsive traits in most of the adult‐onset idiopathic focal dystonias, confirming older observations.^74^, ^75^, ^76^, ^77^

Functional imaging studies disclose similar disruptions of corticostriatal connectivity in movement disorders and psychiatric diseases. The basal ganglia, once considered exclusively concerned with motor control, are now recognized to have a role in psychiatric disorder as well as sensory perception, cognition, and sleep regulation. The Bayesian model^78^ of predictive sensorimotor programming provides a different angle on what used to be taken as evidence of dystonia's inherent psychogenicity. According to the theory, a motor command is coded as a blueprint for sensory feedback expected from the intended action. This helps to explain task‐specificity, the “proprioceptive switch” for dystonic motor output, and the curious *geste antagoniste*. Bayesian ideas have also been applied to functional dystonia, which can be conceived as disordered motor activity driven by abnormal belief—in this context, sensorimotor synaptic networks containing aberrant probability information—residing at an intermediate level within the brain's motor hierarchy.^79^

Compared with most other movement disorders, dystonia is a new conceptual package. Some historical tensions—competing ideas about neurological and psychiatric causation—remain incompletely reconciled. Advances in neuroimaging and cognitive neuroscience have weakened the supposition that dualistic mind‐brain models are useful tools in movement disorder classification. Without this ideological barrier, it may be possible to develop more inclusive biopsychosocial approaches to dystonia.

## Author Roles

1. Research Project: A. Conception, B. Organization, C. Execution; 2. Statistical Analysis: A. Design, B. Execution, C. Review and Critique; 3. Manuscript Preparation: A. Writing the First Draft, B. Review and Critique.

R.E.N.: 1A, 1B, 3A, 3B

D.E.T.: 1A, 1B, 3A, 3B

P.A.K.: 1A, 1B, 3A, 3B

J.E.A.: 1A, 1B, 3A, 3B

## Disclosures

**Ethical Compliance Statement:** We confirm that we have read the Journal's position on issues involved in ethical publication and affirm that this work is consistent with those guidelines.

**Funding Sources and Conflict of Interest:** Rachel E. Newby is supported by a Monash Neurosciences Movement Disorders Fellowship grant. Deborah E. Thorpe was funded in part by the Wellcome Trust [ref: 105624] through the Centre for Chronic Diseases and Disorders (C2D2) at the University of York. Jane E. Alty reports grants from Parkinson's UK, UCB Pharma, Ipsen Ltd., Medtronic, and Merz; honoraria from Allergan; royalties from the Taylor & Francis Group; and stock ownership in Clearsky Medical Diagnostics. Peter A. Kemster reports no sources of funding and no conflicts of interest.

**Financial Disclosures for the previous 12 months:** The authors report no sources of funding and no conflicts of interest.
